# Supplementary material for: Community-Based Interventions to Reduce Child Stunting in Rural Guatemala: A Quality Improvement Model
Source: Int J Environ Res Public Health. 2021 Jan 18;18(2):773. doi: 10.3390/ijerph18020773 (PMC7831302; doi:10.3390/ijerph18020773)
Supplement: Supplementary file 1 [file ijerph-18-00773-s001.pdf]

Supplementary Table 1. Description of community nutrition package components.

Component	Description
Growth monitoring	In-home monitoring, by frontline nutrition workers, of weight and length/height monitoring every month (weekly for children with acute wasting) for all children under 5 years of age. Data are plotted against the World Health Organization child growth standards [1] and shared with caregivers.
Deworming medication	Yearly for all children older than 2 years. Individualized administration for children before 2 years of age, based on input from supervising physicians.
Multiple micronutrient powder	Locally produced product known as "Chispitas": ferrous fumarate (12.5 mg), zinc gluconate (5 mg), retinol acetate (300 mcg), folic acid (160 mcg), and ascorbic acid (30 mg). Daily sachet mixed with food for all children beginning at 6 months of age.
Individualized complementary and responsive feeding education	Individualized in-home counseling sessions led by frontline nutrition workers, provided monthly for 6 sessions for all households with children with stunting under 2 years of age. Counseling sessions use World Health Organization diet recall tools to focus attention on diet diversity (number of food groups consumed per day) and meal frequency (number of time per day that solid-consistency foods are consumed) [2].
Household food rations	Monthly food rations provided for selected households with children exhibiting significant growth faltering and younger than 24 months. Rations include dry black beans, eggs, and fortified infant cereal. On a case by case basis, determined by supervising nutritionist, supplemented with milk formula for young infants whose mothers experience lactational failure, or with therapeutic F-100 formula for children with acute wasting [3].
Medical care	Community health workers trained in Integrated Management of Childhood Illness [4] clinical guidelines for managing acute respiratory and diarrheal illness. Urgent care/episodic care available through referral to public health post or MHA physicians. Vaccines on national schedule provided by public health brigades.

References

[1] World Health Organization. WHO Child Growth Standards. Available at: https://www.who.int/childgrowth/en/ (Accessed 26 November 2020).

[2] World Health Organization. *WHO Policy Perspectives on Medicines-Promoting Rational Use of Medicines: Core Components WHO Policy Perspectives on Medicines*; Geneva, Switzerland: World Health Organization, 2002.

[3] World Health Organization. *Severe Malnutrition: Report of a Consultation to Review Current Literature Nutrition for Health and Development World Health Organization*. Geneva, Switzerland: World Health Organization, 2005.

[4] World Health Organization. Integrated Management of Childhood Illness. Available at: https://www.who.int/maternal_child_adolescent/child/imci/en/ (accessed 20 November 2020).
